# Effectiveness and safety profile of chidamide as maintenance therapy for peripheral T-cell lymphoma: a multicenter real-world study

**DOI:** 10.3389/fonc.2025.1596270

**Published:** 2025-09-02

**Authors:** Wenjing Jiang, Xiyuan Zhang, Wei Song, Hong Xu, Changrong Wei, Juan Wang, Ling Wang

**Affiliations:** ^1^ School of Medcine, Qingdao University, Qingdao, China; ^2^ Department of Hematology, Qingdao Central Hospital, University of Health and Rehabilitation Sciences (Qingdao Central Hospital), Qingdao, China; ^3^ Department of Hematology, The Affiliated Hospital of Qingdao University, Qingdao, China; ^4^ Department of Hematology, Linyi People’s Hospital, Linyi, China; ^5^ Department of Hematology, Qilu Hospital of Shandong University, Qingdao, China

**Keywords:** peripheral T-cell lymphoma, chidamide, maintenance therapy, effectiveness, safety

## Abstract

**Objective:**

Peripheral T-cell lymphoma (PTCL) is characterized by its significant variability and complexity as malignancy. Treatment outcome is poor with conventional chemotherapy. We sought to exploring the effectiveness and safety profile of chidamide monotherapy as maintenance regimen after chemotherapy.

**Methods:**

69 patients for PTCL were included in the study and chidamide was administered as maintenance therapy at a dosage of 15–30 mg twice weekly (biw). The Kaplan-Meier survival analysis was employed to evaluate overall survival (OS) and progression-free survival (PFS).

**Results:**

The average age of participants was 61 years (17-93). The most prevalent pathologic subtype identified was angioimmunoblastic T-cell lymphoma (AITL, 55.1%), and 43.5% (30/69) of patients were classified as intermediate or high-risk cases. Of the patients, 56.5% (n=39) underwent chidamide maintenance therapy after attaining complete response (CR). Over a median follow-up duration of 43.4 months (21.7-98.4), 47.8% of patients attained CR. The median overall survival (mOS) was not achieved, while the median progression-free survival (mPFS) stood at 54.8 months (95%CI, 21.68-72.78). 20% (6/30) of PR patients exhibited CR after chidamide. Individuals who attained CR at baseline demonstrated superior PFS compared to those in PR group. Baseline effectiveness was recognized as an independent prognostic indicator for PFS. Neutropenia was the most common hematologic TRAE, with a 20.3% rate of grade 3/4 events. Dosage modifications were required for 17 patients owing to adverse events, with no fatalities attributed to the treatment reported.

**Conclusion:**

In patients with PTCL, chidamide as a single-agent maintenance treatment demonstrates effectiveness and favorable tolerability, while the remission status prior to initiating maintenance therapy is a key factor influencing treatment outcomes. Notably, the depth of response after induction therapy alone cannot determine the long-term efficacy in PTCL. Maintenance therapy can not only bring more significant benefits to CR patients but also improve the prognosis of PR patients and reduce the risk of recurrence and progression, highlighting the core value of the “induction-maintenance” sequential model. Nevertheless, this study is exploratory, and further verification through prospective studies is still required.

## 1 Introduction

PTCL represents a category of lymphocytic malignancies originating from post-thymic mature T cells or NK cells, known for their significant heterogeneity and aggressive nature. Approximately 10% of non-Hodgkin’s lymphoma (NHL) are classified as PTCL. In Asia, it accounts for about 20-25% of NHL. Frequent variants of PTCL encompass NK/T-cell lymphoma (NKTCL), angioimmunoblastic T-cell lymphoma (AITL), anaplastic large cell lymphoma (ALCL), and peripheral T-cell lymphoma-not otherwise specified (PTCL-NOS). At present, there is no standardized first-line treatment regimen for PTCL except for ALK+ anaplastic large cell lymphoma (ALK+ ALCL). Clinically, the CHOP/CHOP-like chemotherapy regime is commonly introduced as the initial treatment regime. However, treatment outcome is poor with conventional chemotherapy (1).

It has been demonstrated that abnormal epigenetic alterations provide key initiating factors for PTCL recently (2). As a major epigenetic regulator, histone deacetylases (HDAC) can deacetylate lysine residues to condense chromatin structure and ultimately repress transcription of downstream genes (3). As a novel HDAC inhibitor, chidamide triggers G0/G1 cell cycle arrest cell cycle inhibition and apoptosis in tumor cells by enhancing the expression levels of CDK inhibitors and DR6-related apoptotic genes (4). Through the upregulation of SOCS3 and the downregulation of JAK2 and STAT3, it inhibits downstream STAT3-regulated genes, including c-Myc, Bcl-xL, and Mcl-1, promoting cell cycle arrest and apoptosis (5). Chidamide inhibits the activity of HDAC3, HDAC1, HDAC3, and HDAC10 selectively (6), suppressing tumor cell proliferation and triggers tumor cell apoptosis effectively. A prospective study has indicated that chidamide maintenance after first-line therapy has more favorable response rates in PTCL patients who were not suitable for transplantation. The complete response (CR) rate and overall response rate (ORR) were 60.4% and 93.8%, respectively, and the safety profile was manageable (7). In December 2014, It has been authorized by the CFDA as a therapeutic option for relapsed and refractory PTCL (8). To date, the efficiency and safety profile of chidamide as maintenance therapy in the real world have not been established yet. In the study, we examined the effectiveness and safety profile of chidamide as maintenance therapy following chemotherapy for PTCL.

## 2 Patients and methods

### 2.1 Patients

This multicenter, single-arm, retrospective study evaluated the effectiveness and safety of chidamide maintenance therapy in PTCL patients. Data were collected from electronic medical records and telephone follow-ups across multiple centers (June 2016–October 2022).Quality Control Measures:1.Standardized eCRFs ensured uniform diagnosis (WHO 2022 classification), response assessment (Lugano 2014 criteria), and adverse event reporting (CTCAE v5.0);2.Two senior pathologists independently reviewed diagnoses, with unresolved discrepancies adjudicated via multidisciplinary consensus;3.Data underwent dual-entry verification, with third-party expert arbitration for discrepancies;4.Cases with >5% missing baseline data were excluded from primary analysis. Inclusion Criteria: 1. Patients aged 15–95 years (for those over 80 years old and performance status score (ECOG)≤1 was evaluated by the researcher for enrollment);2. Patients were confirmed by histopathology (ALK+ ALCL was excluded);3.Patients who have attained at least PR following induction therapy; Exclusion Criteria: Concurrent hematologic malignancies, active secondary cancers, pregnancy/lactation, drug allergy, NYHA class IV heart failure, or LVEF <40%.

All participants gave their informed consent in accordance with the ethical standards authorized by the hospital’s Ethics Committee. hospital’s Ethics Committee (Approval No.: KY202415401).

### 2.2 Study design and evaluation

Following induction chemotherapy, patients received oral chidamide maintenance therapy at a recommended dosage of 20-30mg twice weekly (with ≥ 3-day intervals between doses). Dose adjustments were permitted based on individual tolerance and treatment response. Effectiveness and safety were assessed every 4-week cycle.

The principal endpoint of the study was PFS: the duration from enrollment to illness progression or the final follow-up. The secondary endpoint of study were OS and event-free survival (EFS). OS: the duration from enrollment to mortality or the final follow-up; EFS: the duration from enrollment to the occurrence of predefined event (such as disease progression, recurrence, death) or the last follow-up.

Effectiveness was assessed according to the 2014 Lugano Response Criteria (9), which were classified as complete response (CR), partial response (PR), stable disease (SD), and disease progression (PD).

Safety evaluation: The criteria for assessing safety include the type, incidence, and severity of adverse events, covering clinical symptoms, abnormal vital signs, and laboratory abnormalities, focusing on grade 3–4 adverse events. The intensity of adverse events was evaluated using the NCI CTCAE v4.03 guidelines.

### 2.3 Statistical analysis

Survival Analysis: Kaplan-Meier curves were plotted to estimate survival distributions, and between-group differences were assessed using the log-rank test. Univariate and multivariate analyses were performed using Binary logistic regression models, with the initiation of maintenance therapy as time zero. Potential prognostic factors were first screened by univariate (selection criterion: P<0.05), and significant variables were subsequently included in the multivariate analysis for adjustment. Results are presented as odds ratios (OR) with 95% confidence intervals (95% CI). Model goodness-of-fit was assessed using the Hosmer-Lemeshow test. A staged variable selection strategy was employed to control for confounding factors. For subgroup analyses, the Bonferroni correction was applied for multiple comparisons (adjusted P=0.05/number of subgroups). A two-sided α level of 0.05 was set as the significance threshold. All statistical analyses were conducted using SPSS 25.0 software.

Safety analysis: The safety analysis is mostly descriptive statistical analysis. The occurrence and level of adverse events (AEs) in this test are described in a list.

## 3 Results

### 3.1 Patient characteristic

**Figure 1:**
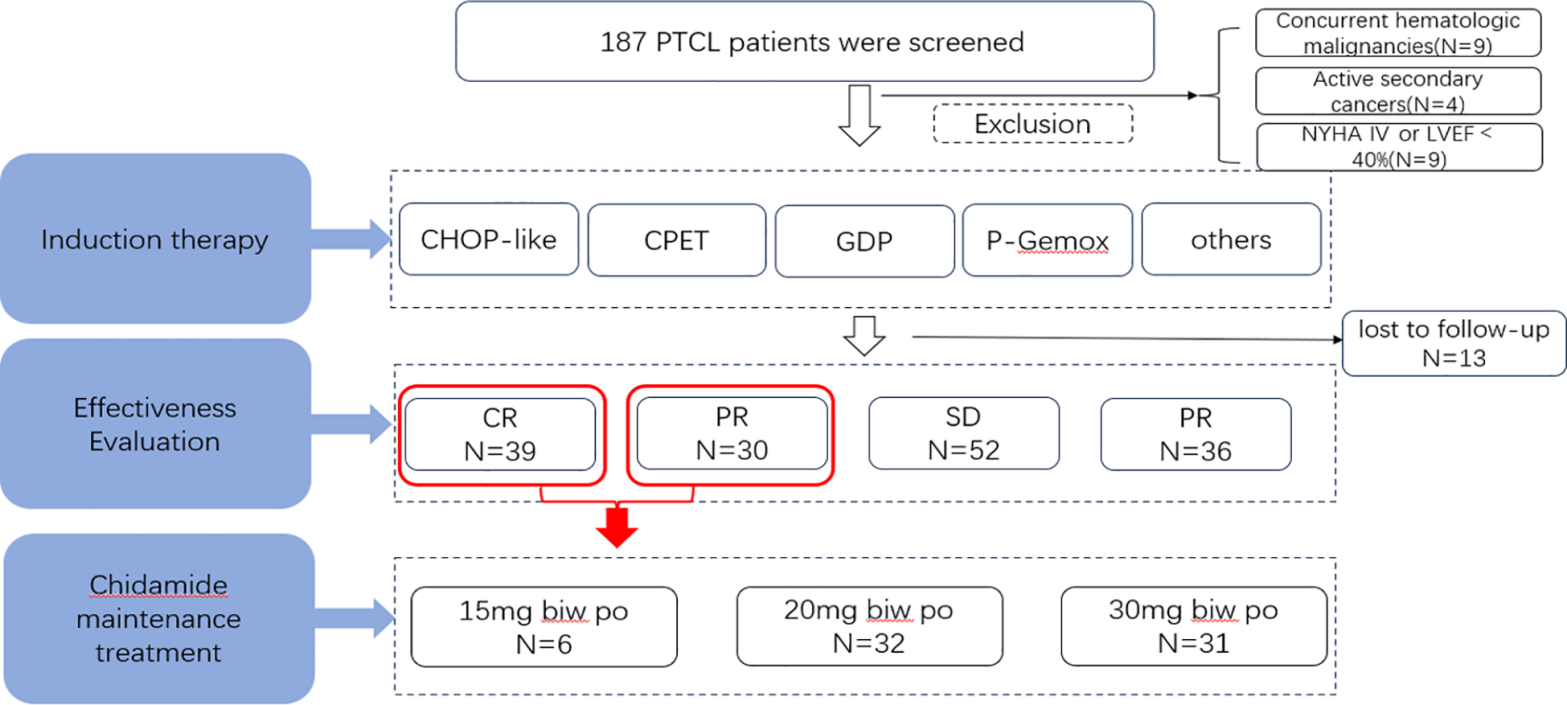
Study design of the enrolled 69 patients.

69 patients were enrolled between June 2016 and October 2022, and 88 patients were excluded due to stable or progressive disease (**Figure 1**). With a median age of 61 years (17-93), 60.9% (42/69) of patients were male, and most of the pathologic subtypes were AITL (55.1%), ALK-ALCL (11.6%), PTCL-NOS (21.7%). Of patients, 43.5% were classified as intermediate or high risk. All received systemic chemotherapy before enrollment, 60.9% (42/69) of patients received second-line or higher treatment, and the remaining 39.1% (27/69) were on maintenance therapy after first-line therapy. 10 patients (14.5%) received hematopoietic stem cell transplantation, comprising 8 patients who received autologous transplants and 2 patients who received allogeneic transplants. 50.7% (35/69) of patients presented with B symptoms, and 94.2% (65/69) had an ECOG score of 0-2. 66.7% (46/69) were staged as III-IV, 91.3% (63/69) had a chidamide maintenance dose of 20–30 mg biw, and only 8.7% (6/69) had a dose reduction at 15 mg biw due to intolerance. Before maintenance therapy with chidamide, all patients achieved CR or PR, with 56.5% (39/69) in CR and 43.5% (30/69) in PR. (**Table 1**).

**Table 1:** The baseline profiles of patients.

Characteristics	Numbers (N=69)	%
Gender		
male	42	60.9
female	27	39.1
Age (median, range)	61 (17-93)	
Pathological subtype		
AITL	38	55.1
PTCL-NOS	15	21.7
ALK-ALCL	8	11.6
NKT	5	7.2
Enteropathy-associated T-cell lymphoma	2	2.9
Cutaneous T-cell lymphoma	1	1.4
Hematopoietic stem cell transplantation		
yes	10	14.5
no	59	85.5
Refractory or relapsed		
yes	42	60.9
no	27	39.1
Baseline effectiveness		
CR/CRu	39	56.5
PR	30	43.5
Ann Arbor stage		
I-II	23	33.3
III-IV	46	66.7
B symptom		
yes	35	50.7
no	34	49.3
ECOG score		
0–2 points	65	94.2
3–4 points	4	5.8
IPI score		
0–2 points	39	56.5
3–5 points	30	43.5
Initial maintenance dose		
15mg biw po	6	8.7
20mg biw po	32	46.4
30mg biw po	31	44.9

### 3.2 Effectiveness

**Figure 2:**
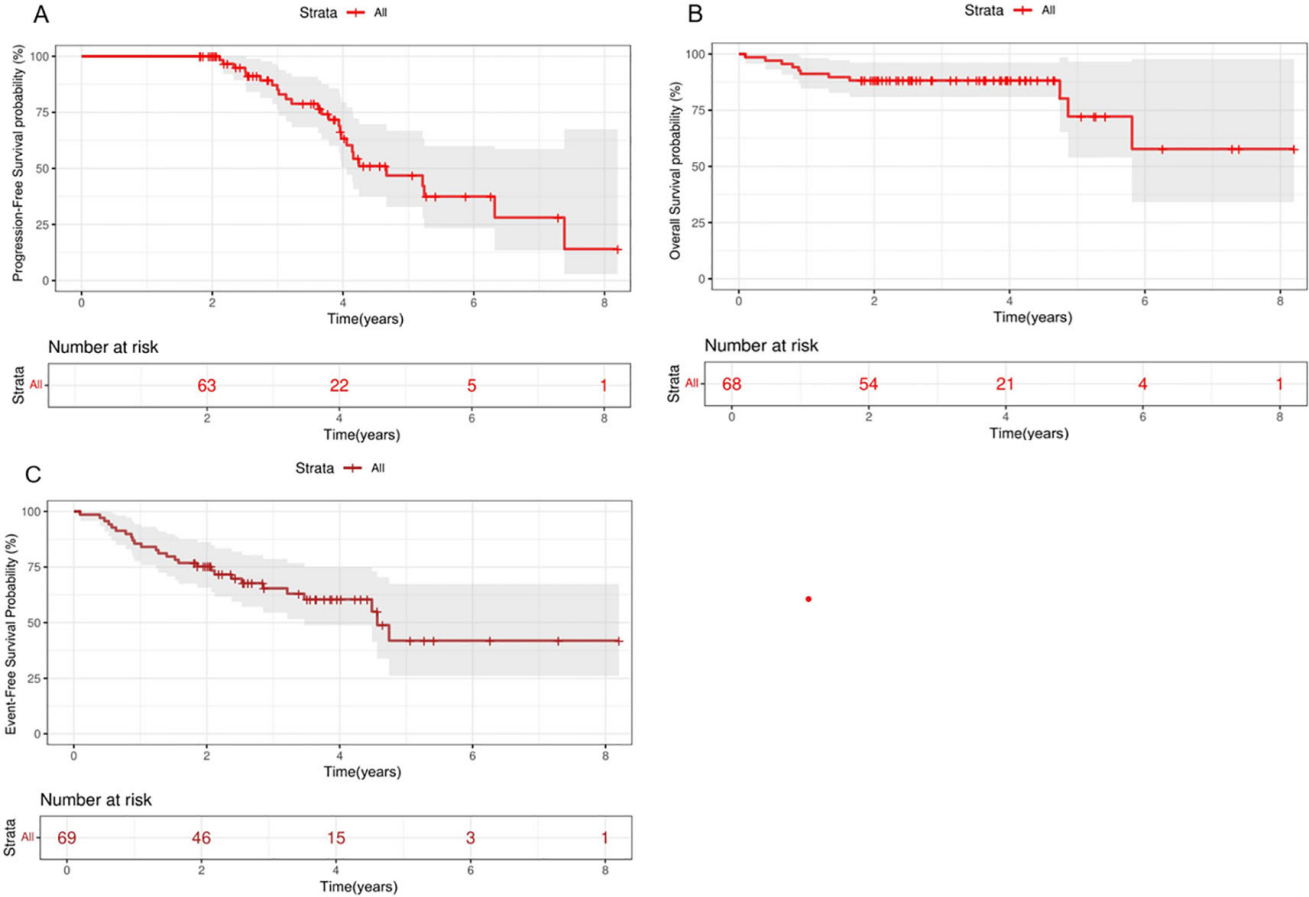
Kaplan-Meier analysis of PFS, OS and EFS in patients with PTCL treated with chidamide maintenance therapy. Survival curves. PFS **(A)**, OS **(B)** and EFS **(C)** in total. OS, overall survival; PFS, progression-free survival; EFS, event-free survival.

Of the 69 patients enrolled, the median follow-up time was 43.4 months (21.7-98.4), more than 91% of participants had a follow-up duration lasting beyond two years. During the follow-up period, 15 patients discontinued the medication because of disease progression, 2 patients discontinued due to drug intolerance (because of neutropenia), 8 patients discontinued for personal reasons, and 1 participant was lost across the follow-up duration. All participants were evaluated for effectiveness, the overall CR rate was 47.8% at the termination of the follow-up stage, and the average duration was 31.4 months. The mOS was not reached, while the mPFS was 54.8 months (95%CI, 21.68-72.78) (**Figures 2A, B**). Disease progression occurred in 37.7% of patients, and 30.8% (12/39) experienced progression in patients for CR and 46.7% (14/30) in patients for PR. Eleven participants died, two of them due to disease progression, one due to cryptococcal infection, and the other due to unknown causes. Among patients with PR, 20% (6/30) achieved CR after receiving chidamide as maintenance therapy. None of these patients had undergone hematopoietic stem cell transplantation, 83.3% (5/6) were over 60 years old, 66.7% (4/6) had previously received induction therapy combined with chidamide (**Table 2**).

**Table 2:** Clinical characteristics of patients with PR-to-CR conversion (n=6).

Patient ID	Gender	Age	Histologic subtype	IPI score	Clinical stage	Relapsed/Refractory	Induction regimen	Chidamide dose	Time from PR to CR (months)
1	Male	72	AITL	1	I	NO	GDP×9 cycles	20mg	11.4
2	Female	40	PTCL-NOS	1	I	NO	CHOPE×4 cycles	15mg	2.3
3	Male	61	AITL	1	II	Yes	CHOP×6 cycles →ycl ×2 cycles	30mg	2.7
4	Female	62	PTCL-NOS	3	III	Yes	CHOP×3 cycles →Gemox×4 cycles	30mg	6.1
5	Male	69	ALK-ALCL	2	II	NO	CPET×8 cycles	20mg	4.2
6	Female	68	AITL	2	III	Yes	CHOP×6 cycles →ycle×6 cycles	20mg	4.8

**Figure 3:**
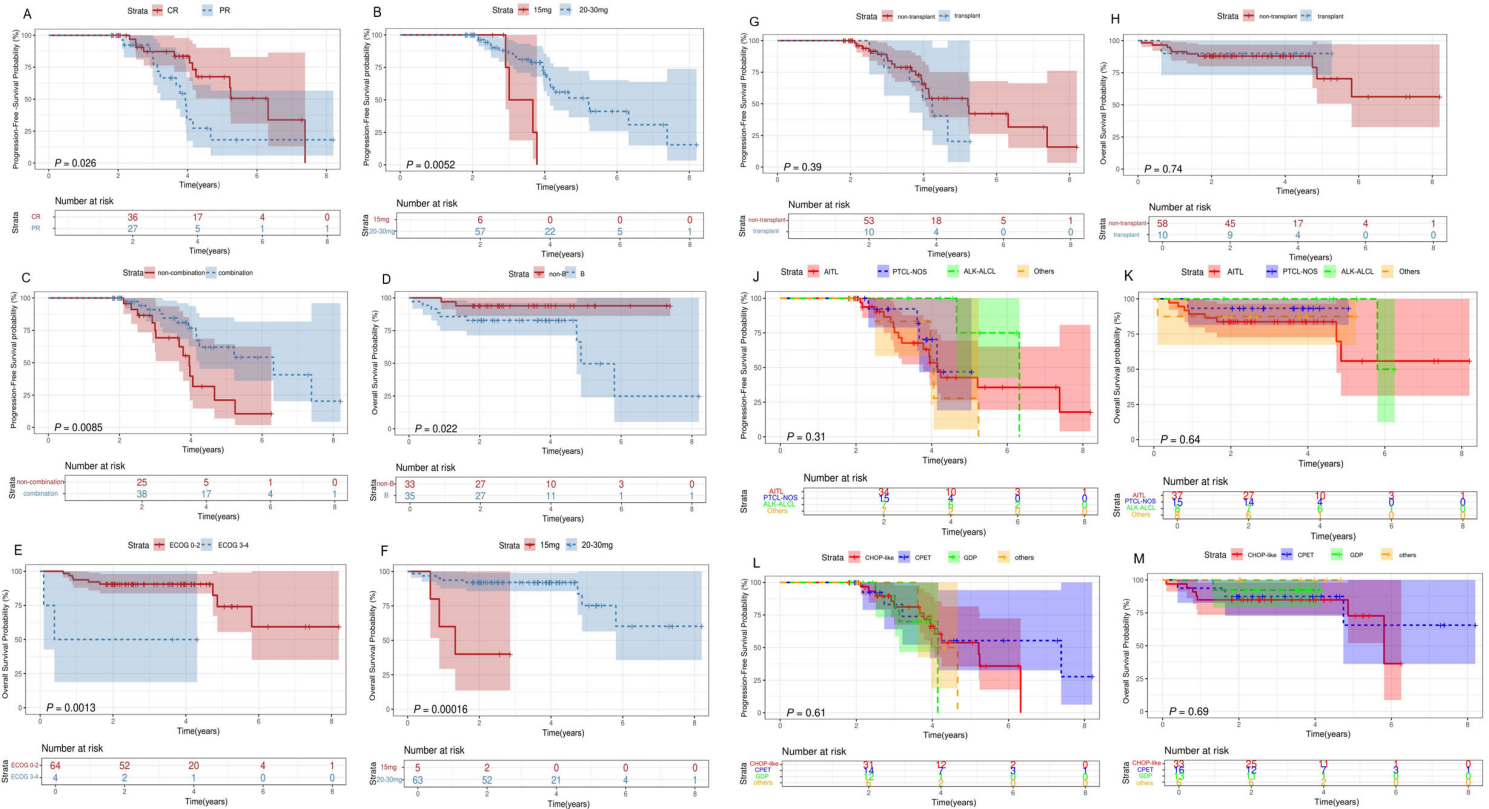
Survival analysis for patients with PTCL receiving chidamide maintenance treatment. Survival curves. **(A)** Comparisons of the PFS between PTCL patients who achieve CR or PR before maintenance therapy. **(B-F)** Comparisons of the OS **(F)** and PFS **(B)** between patients treated with 20mg/30mg of chidamide. **(C)** Comparisons of the PFS between patients treated with induction therapy in combination with and without chidamide. **(D)** Comparisons of the OS between PTCL patients with and without B symptoms. **(E)** Comparisons of the OS between patients with ECOG 0-2 and 3-4. Comparisons of the OS **(H)** and PFS **(G)** between patients with and without transplantation before maintenance treatment. **(J, K)** Comparisons of the OS **(K)** and PFS **(J)** between various histopathologies. **(L, M)** Comparisons of the OS **(M)** and PFS **(L)** between various induction therapy regime. OS, overall survival; PFS, progression-free survival; ECOG, Eastern Cooperative Oncology Group; CR, complete response; PR, partial response.

**Figure 4:**
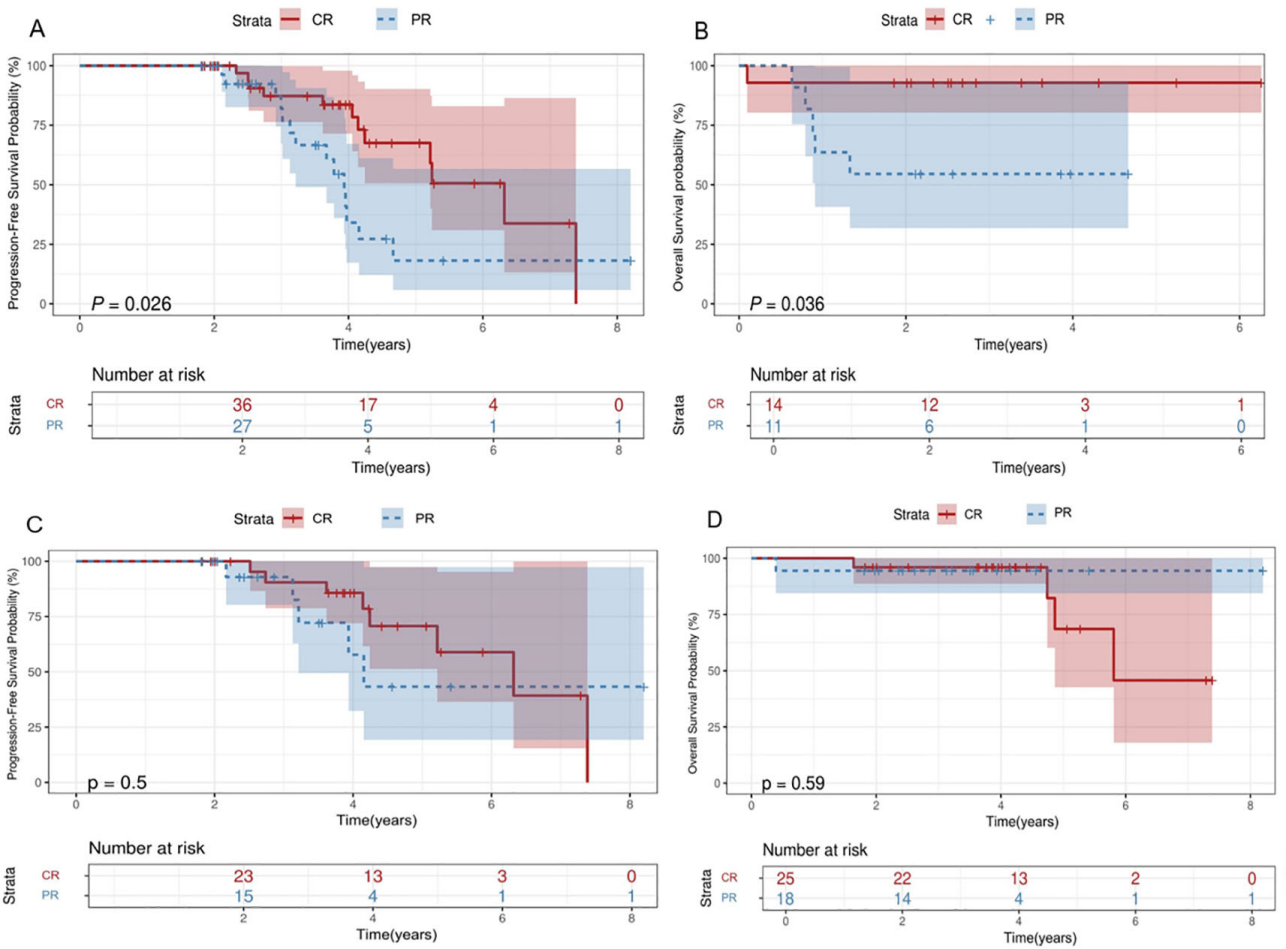
Subgroup survival analysis. Survival curves. **(A, B)** Comparisons of the OS **(B)** and PFS **(A)** between patients achieve CR/PR during induction treatment without chidamide. **(C, D)** Comparisons of the OS **(D)** and PFS **(C)** between patients achieve CR/PR during induction treatment with chidamide OS, overall survival; PFS, progression-free survival; CR, complete response; PR, partial response.

The 2-year PFS, OS, and EFS rates for the entire patient cohort were 89.3% (95% CI,79.3%-100%), 88.2% (95%CI, 81.3%-91.7%) and 75.3%(95CI,67.8%-85.4%), respectively. The 5-year PFS, OS, and EFS rates for the entire patient cohort were 47.4% (95%CI,32.3%-66.9%), 72.8%(95%CI,54.6%-90.8%) and 41.9%(95CI,25.9%-67.5%), respectively (**Figures 2A–C**). Patients who achieved CR at baseline exhibited a higher PFS rate than those in PR group (P=0.026) (**Figure 3A**). Elevated doses of chidamide were associated with better PFS and OS outcomes compared to the 15 mg dosage (**Figures 3B, F**). Chidamide-based induction treatment also improved PFS over induction therapy alone (P=0.0085) (**Figure 3C**). The OS of participants without B symptoms was superior to those with B symptoms (P=0.022) (**Figure 3D**). The OS of patients with ECOG score 0–2 was also superior to those with ECOG score 3-4 (P=0.0013) (**Figure 3E**). HSCT did not significantly influence the effectiveness of chidamide maintenance therapy (**Figures 3G, H**). No notable differences were detected among subgroups categorized by histopathology or induction therapy strategies (**Figures 3J–M**). For subgroup analyses, in patients treated with induction therapy without chidamide, CR patients before maintenance treatment had superior PFS and OS than those achieving PR (**Figures 4A, B**). No statistically differences in PFS and OS were observed between CR and PR patients who received chidamide during their initial induction therapy (adjusted P=0.025) (**Figures 4C, D**).

### 3.3 Univariate and multivariate analysis

Univariate analysis indicated that pretreatment regime, baseline remission status and chidamide dose influenced PFS, while ECOG, pretreatment regime and chidamide dose were linked to OS. Multivariate analysis indicated that pretreatment regime and baseline remission status were independent predictors of PFS (HR=0.225, P=0.026; HR=4.309, P=0.036) and ECOG and chidamide dose were independent predictors of OS (HR=12.749, P=0.036; HR=18.448, P=0.010) (**Table 3**).

**Table 3:** Univariate and multivariate analysis of factors correlated with PFS and OS. .

Parameter	PFS at 2 years				OS at 2years			
	Univariate analysis		Multivariate analysis		Univariate analysis		Multivariate analysis	
	OR (95% CI)	P	OR (95% CI)	P	OR (95% CI)	P	OR (95% CI)	P
Age								
≤60y vs >60y	0.825 (0.275-2.478)	0.732			1.400 (0.319-6.137)	0.655		
Ann-Arbor stage								
I-II vs III-IV	1.575 (0.508-4.882)	0.431			0.429 (0.096-1.905)	0.266		
B symptoms								
No vs Yes	0.648 (0.214-1.964)	0.443			3.207 (0.599-17.181)	0.174		
ECOG score								
0–2 vs 3-4	3.333 (0.432-25.716)	0.248			9.667 (1.146-81.555)	**0.037**	12.749 (1.187-136.975)	**0.036**
IPI								
0–2 vs 3-5 HSCT	0.450 (0.139-1.459)	0.183			0.733 (0.161-3.350)	0.689		
No vs Yes	1.378 (0.314-6.049)	0.671			0.810 (0.089-7.393)	0.851		
Baseline effectiveness								
CR/CRu vs PR	4.533 (1.380-14.893)	**0.013**	4.309 (1.100-16.885)	**0.036**	4.826 (0.897-25.967)	0.067		
Pretreatment lines								
First-line vs Second-line and above	2.125 (0.700-6.452)	0.183			1.609 (0.366-7.070)	0.529		
Pretreatment regime*								
No vs Yes	0.221 (0.069-0.707)	**0.011**	0.225 (0.061-0.833)	**0.026**	0.154 (0.028-0.837)	**0.030**	0.324 (0.048-2.203)	0.249
Pathological subtype								
PTCL-NOS vs AITL	1.630 (0.384-6.922)	0.508			2.710 (0.298-24.678)	0.376		
Other types vs AITL	1.698 (0.546-5.275)	0.360			2.806 (0.524-15.036)	0.228		
Maintenance dose								
20mg-30mg vs 15mg	7.692 (1.267-46.709)	**0.027**	1.951 (0.247-15.435)	0.526	17.400 (2.334-129.721)	**0.005**	18.448 (1.985-171.499)	**0.010**

OS, overall survival; PFS, progression-free survival; CI, confidence interval; HSCT, hematopoietic stem cell transplantation; HR, hazard ratio * whether induction treatment were combined with chidamide or not. Bold values indicate statistically significant differences (P≤0.05).

### 3.4 Safety

Chidamide maintenance treatment was well tolerated. Neutropenia was the most frequently AE during maintenance treatment, with an incidence of grade 3/4 AE at 20.3% and anemia (n=13, 18.8%) with only 2.9% classified as grade 3/4. 5 patients (35.7%) had febrile neutropenia. The most frequently non-hematologic AEs were gastrointestinal symptoms and malaise, with no grade 3/4 AEs observed. Seventeen enrolled patients had dose adjustments due to adverse reactions, and no fatalities associated with treatment were observed throughout the study (**Table 4**).

**Table 4:** Adverse events in maintenance treatment with chidamide.

Toxicity	Grade 1 N	Grade 2 N	Grade 3 N	Grade 4 N	Total N(%)	Grade≥3 N(%)
Thrombocytopenia	6	10	8	2	26 (37.7)	10 (14.5)
Anemia	8	3	1	1	13 (18.8)	2 (2.9)
neutropenia	6	12	10	4	32 (46.4)	14 (20.3)
leukopenia	9	11	5	2	27 (39.1)	7 (10.1)
Elevated transaminases	0	1	0	1	2 (2.9)	1 (1.4)
gastrointestinal symptoms	9	1	0	0	10 (14.5)	0 (0)
Diarrhea	2	1	0	0	3 (4.3)	0 (0)
Fever	1	0	0	0	1 (1.4)	0 (0)
Weakness	10	0	0	0	10 (14.5)	0 (0)
Edema	1	0	0	0	1 (1.4)	0 (0)

## 4 Discussion

Previous studies have shown that PTCL recurrence after induction therapy leads to poor survival, with the average survival of lower than 4 years mostly (10). Thus, there is an urgent need for developing a new treatment method for preventing the recurrence of PTCL.

Many studies have shown dysregulation of epigenetic modifications in PTCL patients (11). Histone acetylation, as the most common modification, plays a significant regulatory role in morphogenesis, proliferation, angiogenesis, and apoptosis, which is closely related to cancer development and progression (12). Therefore, histone deacetylase (HDAC) can be a potential therapeutic target. Importantly, a unique interaction pattern of HDACs with chidamide makes it more stable at the catalytic site, which maintains a relatively high level of histone acetylation within 72 hours after administration (13).

Chidamide, as an HDAC inhibitor, can regulates gene expression by modulating histone acetylation, inhibiting tumor cell proliferation, and epigenetic inducing apoptosis. It also plays a role in immune-mediated anti-tumor effects across various cancers. Liu et al. (14) used single-cell technology to reveal immune heterogeneity in PTCL bone marrow involvement. They identified a highly heterogeneous immune cell population, particularly an increase in exhausted and regulatory T cells. The PTCL bone marrow microenvironment also exhibited significant immunosuppressive traits, including impaired effector T-cell function and upregulated of immune checkpoint molecules like PD-1 and CTLA-4. These factors likely contribute to immune evasion, highlighting the need to improve the tumor microenvironment and restore anti-tumor immunity in PTCL patients. Wei et al. (15) demonstrated that chidamide reshapes the tumor microenvironment to enhance anti-tumor immunity. It facilitates the migration of lymphocytes, monocytes, neutrophils, and dendritic cells, while enhancing chemotactic gene expression in circulating PD-1(+) cells. Chidamide also upregulates genes related to lymphocyte chemotaxis, TNF signaling, and IFN-γ responses, facilitating lymphocyte recruitment and the recovery of exhausted CD8+ T cells. Clinical studies have combined chidamide with immune checkpoint inhibitors to overcome resistance and amplify host immune responses, showing promising effectiveness in PTCL treatment and offering a novel therapeutic approach. This provides a theoretical foundation for the maintenance treatment of chidamide.

Some studies have shown that chidamide maintenance therapy may result in the conversion from PR to CR and from SD to PR or CR, ultimately achieving durable remission. Chidamide monotherapy maintenance also can reduce relapse and progression after HSCT (7). In addition, Wei et al. (16) found that PTCL patients receiving maintenance therapy with chidamide had better PFS and OS than those who did not undergo maintenance therapy. A multi-center, phase 2 prospective study (NCT02987244) found that in PTCL patients after remission, chidamide maintenance as a single-agent therapy showed significantly improved PFS and OS compared to observation (17). Therefore, maintenance therapy with chidamide represents a hopeful therapeutic option for PTCL.

Romidepsin, a first-generation HDAC inhibitor, plays a crucial role in maintenance therapy. Foss et al. found that for patients with R/R PTCL, even if they fail to achieve CR or PR, maintenance therapy with Romidepsin can still result in long-term stability (18). PD-1 blockade has shown effectiveness in patients for PTCL. Consequently, Merriill et al. (19) conducted a phase 2, multicenter research to explore the effectiveness of pembrolizumab for PTCL in first remission after ASCT, which proved to be both practical and promising, with a well-tolerated profile.

Chidamide was utilized for maintenance following induction therapy in our research. Over a follow-up duration of 43.4 months (21.7-98.4), the mOS was not reached in all patients, and the mPFS was 54.8 months (95%CI, 21.68-72.78). Compared to previous studies, our research indicates that maintenance therapy with chidamide can achieve long-term remission in PTCL and enhance the effectiveness of induction therapy. Although some patients (N=30, 43.5%) only achieved PR in the induction therapy, six patients (20%) benefited from maintenance therapy, achieving better outcomes. This suggests that continuous maintenance therapy with chidamide may lead to conversion from PR to CR, ultimately achieving long-term remission. Maintenance strategy with chidamide monotherapy significantly reduces the risk of relapse and progression due to inadequate depth of remission before the administration of chidamide. Wei et al. (16) observed that the CHOPE regime combined with chidamide improved the CR rate in untreated PTCL patients. However, higher CR rate did not lead to a prolonged duration of response, and no significant PFS difference was observed between chidamide maintenance and non-maintenance groups. In our study, we identified that among patients receiving C-X or X induction therapy, 69 achieved CR or PR, while 88 experienced PD or SD, resulting in an overall CR rate of 43.9%. After maintenance therapy, the overall CR rate increased to 47.8%.

Furthermore, our study analyzed the influence of maintenance therapy in CR and PR patients. We observed that patients who achieved CR in induction therapy showed better effectiveness compared to those with PR. Both univariate and multivariate analyses showed that CR correlates with improved PFS. These data suggest that the remission status prior to maintenance therapy plays a crucial role in the treatment effectiveness.

López-Guillermo et al. (20) carried out a study on 174 PTCL participants treated with therapeutic regimens, using univariate and multivariate analyses to evaluate the prognostic importance of different predictors. In univariate analysis, factors such as age ≥65 years, ECOG score ≥2, the occurrence of B symptoms, bone marrow infiltration, and higher Ann Arbor stage, and intermediate/advanced-risk IPI were associated with poor prognosis. Multivariate analysis indicated that the manifestation of B symptoms and intermediate/high-risk IPI were independent prognostic factors. Similarly, our results showed that ECOG score 0–2 was linked to better prognosis. Wang et al. (21) found that patients in the chidamide combination therapy had a higher PFS compared to those in the traditional chemotherapy. Additionally, the multivariate analysis showed that initial treatment approach was an independent predictor influencing PFS in PTCL. Standard chemotherapy in combination with chidamide significantly prolonged PFS in those newly diagnosed with PTCL, particularly in patients with elevated IPI scores. Wang et al. (22) also found that chidamide in combination with chemotherapy had significantly better PFS for high-risk, second-line, and CD30-negative patients under 60 years of age compared to traditional chemotherapy and targeted therapy. Our study also found that chidamide combination therapy showed better PFS for PTCL patients. And multivariate analysis revealed that pretreatment regime and baseline remission status served as independent predictors of PFS. However, no statistically differences in PFS and OS were observed between CR and PR patients who receive chidamide or not during their initial induction therapy. Without maintenance, CR and PR patients exhibit comparable outcomes, implying that induction response depth alone does not guarantee long-term disease control. With maintenance, CR patients achieve significantly better PFS than PR patients, indicating that maintenance therapy selectively benefits those with deeper initial responses, further emphasizing the importance of post-induction strategies.

Gkotzamanidou et al. (23) found that ASCT functions as an initial consolidation approach for aggressive lymphomas and a therapeutic option for patients with relapsed or refractory disease. Park et al. (24) found that for patients diagnosed with nodal PTCL who achieved PR1, ASCT as consolidation therapy improved PFS and OS significantly, particularly in PTCL subtypes with poorer prognosis. Huang et al. (25) found that maintenance therapy after transplantation reduced the relapse and improved OS and PFS in PR patients significantly. However, our study showed no statistically difference in PFS and OS between the non-transplant and transplant groups. Therefore, whether transplant patients need further chidamide maintenance therapy needs further investigation. This also indicates that chidamide monotherapy as maintenance therapy may counteract the effects of transplantation in patients. Consequently, it offers a novel maintenance strategy for patients who are unable to undergo HSCT due to personal or disease-related reasons. Additionally, our study showed that 20–30 mg doses of chidamide maintenance therapy has survival benefits in PTCL patients compared to 15mg dose, suggesting that maintenance therapy with high-dose chidamide may be more effective.

In this study, all patients tolerated maintenance therapy after chemotherapy. Although the majority of patients experienced grade 3–4 adverse reactions, which were hematologic toxicities associated with chidamide, they recovered with supportive therapy and continued maintenance therapy in this study, the study reported no treatment-related mortality, indicating that the safety is manageable.

To prevent the risk of PTCL recurrence, various maintenance strategies are being actively explored, **Table 5** below summarizes the findings from previous studies and our study, highlighting the differences in outcomes. In conclusion, in patients with PTCL, maintenance therapy using chidamide alone is not only effective but also well-tolerated. Moreover, the pre-maintenance remission status significantly impacts the treatment’s success. Notably, the depth of response after induction therapy alone cannot determine the long-term efficacy in PTCL. Maintenance therapy can not only bring more significant benefits to CR patients but also improve the prognosis of PR patients and reduce the risk of recurrence and progression, highlighting the core value of the “induction-maintenance” sequential model.

**Table 5:** Comparison of maintenance therapy: other studies vs our study.

Year	Author	Type	n	Pre-treatment	Treatment	Conclusion
2023	Stuver (26)	a phase II study	39	Patients underwent six cycles of standard-dose CHOEP therapy in combination with 10mg of lenalidomide.	High-dose therapy with auto-HST rescue(n=16), or lenalidomide(n=10)	Among patients Received lenalidomide and underwent HDT/ASCR, and received neither maintenance nor HDT/ASCR, 2-year PFS was 56% (95%CI, 20%-81%) vs 81% (95%CI, 52%-94%) vs 100% (95%CI, 100%-100%), respectively.
2023	Merriill (19)	a phase 2 study	21	Patients receive ASCT after the first remission	Anti–PD-1 monoclonal antibody pembrolizumab	The 18-month PFS estimate was 83.6% (95%CI, 68%-100%) and the OS was 94.4% (95%CI, 84%-100%).
2024	Jiang (Me)	a retrospective single-center study	69	Patients receive induction therapy including CHOP-like regime, CPET, GDP, P-Geomx and others	Chidamide	The overall CR rate was 47.8%, the 2-year PFS and OS rates were 89.3% (95% CI,79.3%-100%) and 88.2% (95%CI, 81.3%-91.7%).

As a retrospective study, the present research is potentially subject to bias, particularly regarding patient selection and uncontrolled confounding factors. Additionally, due to the relative rarity of PTCL and the limited number of clinically accessible cases, the small sample size may compromise statistical power. Given these methodological limitations, this study is positioned as an exploratory investigation, aiming to provide preliminary evidence-based insights into the clinical challenge of maintenance therapy for PTCL. Further multicenter randomized or prospective research is required to investigate the therapeutic potential of chidamide as maintenance therapy in PTCL.

## Data Availability

The raw data supporting the conclusions of this article will be made available by the authors, without undue reservation.
